# Deaths averted: An unbiased alternative to rate ratios for measuring the performance of cancer screening programs

**DOI:** 10.1177/09691413231215963

**Published:** 2023-11-21

**Authors:** Wilber Deck, James A Hanley

**Affiliations:** 1 Direction de santé publique, Gaspé, Quebec, Canada; 2 Department of Epidemiology, Biostatistics and Occupational Health, McGill University, Montreal, Quebec, Canada

**Keywords:** Trials, meta-analyses, cancer mortality, lung cancer screening, dilution bias, risk difference

## Abstract

**Introduction:**

Screening trials and meta-analyses emphasize the ratio of cancer death rates in screening and control arms. However, this measure is diluted by the inclusion of deaths from cancers that only became detectable after the end of active screening.

**Methods:**

We review traditional analysis of cancer screening trials and show that ratio estimates are inevitably biased to the null, because follow-up (FU) must continue beyond the end of the screening period and thus includes cases only becoming detectable after screening ends. But because such cases are expected to occur in equal numbers in the two arms, calculation of the difference between the number of cancer deaths in the screening and control arms avoids this dilutional bias. This difference can be set against the number of invitations to screening; we illustrate by reanalyzing data from all trials of tomography screening of lung cancer (LC) using this measure.

**Results:**

In nine trials of LC screening from 2000 to 2013, a total of 94,441 high-risk patients were invited to be in screening or control groups, with high participation rates (average 95%). In the older trials comparing computed tomography to chest X-ray, 88,285 invitations averted 83 deaths (1068 per death averted (DA)). In the six more recent trials with no screening in the control group, 69,976 invitations averted 121 deaths (577 invitations per DA).

**Discussion:**

Screens per DA is an undiluted measure of screening's effect and it is unperturbed by the arbitrary duration of FU. This estimate can be useful for program planning and informed consent.

## Introduction

In trials of cancer screening, the benefits have traditionally been measured by calculating the rate ratio (RR) of specific cancer mortality, at some follow-up (FU) time several years after the end of the active screening period. For instance, nine trials of low-dose^a^ computed tomography (CT) screening for lung cancer (LC) have been conducted, all analyzed using what was effectively a RR or a hazard ratio (HR), i.e. the LC death rate among persons invited to screening divided by the rate among persons in the control group. In all trials, rates were calculated as the number of LC deaths divided by the person-years (PYs) over the entire FU period, consisting of both the years during which screening was performed and additional years of FU after the final round of screening.

Researchers reporting the results of the largest of these trials, the National Lung Screening Trial (NLST)^1^ noted that “patients in whom cancer did not develop until after the last scheduled screen might not have benefited from the trial screenings; therefore, deaths in such patients would only serve to add noise to the estimate, roughly an equal number of deaths in each arm. Therefore, in analyzing these data, we have used … the difference in LC deaths across arms in addition to the rate ratio.”^1^ They estimated that the “difference in the number of patients dying of LC (per 1000) across arms was 3.3, translating into a NNS [number needed to screen] of 303.” UK Lung Screening Trial (UKLS) researchers,^2^ in a meta-analysis pooling the results of all nine trials, estimated that CT screening in persons with a history of smoking could reduce LC mortality by 16%. They acknowledged that “…the most recent reported results include deaths from LCs diagnosed after the screening phase…While this does not affect the absolute benefit, it dilutes the relative effect of the intervention, conservatively biasing the RR of LC mortality toward unity.”^2^ In a letter responding to this report we suggested that the use of rate difference, as a summary statistic, might be preferable, since “Including the additional FU, where no benefit is expected, attenuates the RR, but doesn’t change the rate difference.”^3^

However, other authors of trial reports or meta-analyses^4–8^ have not presented statistics based on LC mortality differences. We thus present here a more thorough explanation of why the use of RRs or HRs understates the potential benefit of screening. We propose alternative statistics based on the absolute rate difference, i.e. the absolute number of LC deaths averted (DA), expressing this as the number of screening invitations required to avert one LC death, using the most recent data from all nine CT screening trials.

## Methods

After the last round of screening, new cases of cancer will become screen detectable in both screening and control groups but cannot be identified early because screening has ended. These post-screening cases constitute “noise”, obscuring the “signal” from cases treated early because they had become detectable during the active screening program. In a RR analysis, this noise cannot be eliminated, because there is no way of knowing whether an individual case in the control group was one that would have been screen detectable during the screening period, or not. This noise from late LC deaths will push the RR towards unity, since they contribute to the numerator and the denominator.^9^ But importantly, because they tend to occur in equal numbers in both groups, they will not influence the rate difference.^3^ This is illustrated in successive reports from the largest CT trial, the NLST, summarized in Table 1.

**Table 1.** Mortality data at different points of FU, NLST.

FU until	Median FU (years)	LC deaths, control arm (*c*)	LC deaths, screening arm (*s*)	Rate ratio (95% CI^b^)	Additional LC deaths since initial FU (*n_c_*, *n_s_*)	Absolute difference (*c-s*)
15 Jan 2009^a^	5.5	443	356	0.80 (0.73–0.93)	—	87
31 Dec 2009^b^	6.5	552	469	0.84 (0.75–0.95)	*n_c_* = 109, *n_s_* = 113	83
31 Dec 2015^c^	12.3	1236	1147	0.92 (0.85–1.00)	*n_c_* = 793, *n_s_* = 791	89

FU to the end of 2015 for 97.8% of participants; FU for the others was until the end of 2009. FU: follow-up; LC: lung cancer; CI: confidence interval. ^a^NLST, 2011.^10^ ^b^Pinsky, 2013.^11^ ^c^NLST, 2019^1^.

Three rounds of screening took place from year 0 to year 2, followed by 3.5, 4.5 and 10.3 years of further FU. In the 7 years of additional FU between 2009 and 2015, the number of LC DA by NLST screening changed by just 2 (89 instead of 87), and yet the ratio of death rates converged towards unity, going from 0.80 to 0.92. LC deaths detected after 5.5 years of FU occurred in equal numbers in the screening and control groups, showing that screening had no effect beyond 5.5 years; further FU only added deaths from cases not influenced by screening or becoming screen detectable only after screening had ended. With about 100 LC deaths occurring every year in the control group, one can imagine a hypothetical additional 10 years of FU might show an additional 1000 deaths in each group. At that point, the number of DA would remain at 89, but the RR would continue approaching 1.0, going from 1147/1236  =  0.92 to 2147/2236  =  0.96.

Potential solutions to this problem include truncating the FU included in the analysis, as attempted in one trial report,^1^ but this involves choosing an arbitrary cut-off on a timeline where there is continuous change from no effect to partial effect to full effect and then back to partial effect and no effect. Another is the approach taken by Duffy et al.^12^ only counting deaths from cancers that came to light within a window that ends some selected number of years after the last screen, but this too involves an arbitrary cut-off. Conducting a trial with a substantial number of screens over many years of FU would largely solve the problem, since most of the FU would be pertinent to screening. However, such trials would be difficult and costly to perform, and a decades-long delay before publication would tend to render results irrelevant to evolving practice. Our proposal is to use the rate difference in analyzing the existing short trials. Because PY denominators in screening and control groups are never identical (although they were similar in most LC trials), we refine the technique illustrated in Table 1 (which simply used the difference in numbers of deaths) by adjusting for the small differences in numbers of persons in each arm of the trial. We do this by calculating the difference between the number of deaths that would have been expected in the screening group if control group LC mortality rates applied, and the number of deaths observed in the screening group.

We can treat the number of cases observed in the screening group (O_s_) as a realization of a Poisson random variable, and so its estimated variance var is also equal to O_s_, since the variance of a Poisson random variable equals its mean. The expected number of cases in the screening group E_s_  =  O_c_ × (FU_s_/FU_c_) is just a slight alteration of the observed number O_c_ in the control arm, necessitated by the fact that the PYs of FU in the two arms, FU_c_ and FU_s,_ were not identical. Its sampling variation can be modeled as if it were a realization of another independent Poisson random variable, with estimated variance var  =  E_s_. Since the variance of the difference of two independent random variables is the sum of their variances, the variance associated with the number of DA, DA  =  E_s_–O_s_, will be the sum of the variances, namely E_s_  +  O_s_, and so its standard error is the square root of this: (E_s_  +  O_s_)^1/2^. A 95% margin of error can be calculated as 1.96 × (E_s_  +  O_s_)^1/2^.

Beyond the time where benefit has been observed, the benefit in DA (E-O) will remain fixed, but the numbers E and O will continue to increase, so the square root of their sum will also increase indefinitely as FU time increases.

Every case that becomes detectable during the screening period offers an opportunity for averting a LC death, so the number of cases that could benefit from screening is expected to vary directly with the underlying incidence rate of cancers. Because trials recruit populations with different criteria for admission (primarily regarding age and smoking history), the rate difference measured by each trial would be expected to vary directly with the underlying risk of LC. Pooling trials with populations that have different risk levels thus requires adjusting for the risk of LC cancer in each trial. This risk can be estimated by the incidence rate observed in the control group that is unaffected by CT screening. We estimate LC risk from the trials which included no chest X-ray (CXR) screening in the control group, since the incidence rate in a group undergoing CXR screening is much higher than the rate of symptomatic cancers.

## Results

Protocols and participation in the nine screening trials are summarized in Table 2. The two trials from the USA were the pilot Lung Screening Study (LSS)^13^ and the subsequent full NLST,^1^ with similar designs using a contrast between CT screening in the index group and CXR screening in the control group. Although CXR screening had not been shown to be effective in reducing LC mortality in previous trials, it may have enough effectiveness to lessen the gains observed from CT screening compared to no screening at all. In the Detection and Screening of Early Lung Cancer with Novel Imaging Techniques and Molecular Assays (DANTE) trial,^14^ baseline CXR and sputum screening were done in both screening and control groups, with subsequent rounds including a clinical exam in the control group. These earlier trials weigh heavily in meta-analyses, accounting for 56% of all invitations in the nine trials.

**Table 2.** Screening protocols of published low-dose CT trials.

	Control		Screening						
Study	Diagnostic procedure	Persons	Recruitment	Rounds	Interval (months)	Persons	Invitations	Screens performed	Particip. rate (%)
LSS	CXR, each round	1658	Sept 2000 – Nov 2000	2	12	1660	3027	2930	96.8
DANTE	baseline CXR and sputum^a^	1186	Mar 2001 – Feb 2006	5	12	1264	6178	5967	96.6
NLST	CXR, each round	26,730	Aug 2002 – Apr 2004	3	12	26,722	79,080	75,126	95.0
**All** CT vs CXR		29,574	2000–2006	2–3	12	29,646	88,285	84,023	**95** **.** **2**
NELSON	nil	7892	Dec 2003 – Jul 2006	4	I1:12; I2:24; I3:30	7900	28,455	27,051	95.1
DLCST	^b^	2052	1 Oct 2004 – 31 Mar 2006	5	12	2052	10,260	9800	95.5
ITALUNG	nil	1593	2004–2006	4	12	1613	6386	5328	83.4
MILD^c^	nil	1723	Sept 2005 – Jan 2011	4 & 7	12 & 24	2376	13,008	12,375	95.1
LUSI	nil	2023	23 Oct 2007 – 11 Apr 2011	5	12	2029	9886	9405	95.1
UKLS	nil	1981	Oct 2011 – Feb 2013	1	dna	1987	1981	1954	98.6
**All** CT vs no CXR		17,264	2003–2013	1–7	12–30	17,957	69,976	65,913	**94**.**2**
**Total**		46,838	0	1–6	12–30	47,603	158,261	149,936	**94**.**7**

dna: does not apply; Particip.: participation; LSS: Lung Screening Study. ^a^Baseline postero-anterior chest X-ray (CXR) and sputum cytology were performed in control and screening groups. ^b^Annual clinical exam and sputum cytology were conducted in control and screening groups. ^c^MILD: 1186 participants screened annually, 1190 biannually.

Among the six European trials conducted subsequently, none used radiographical screening in the control group. In the Danish Randomized Lung Cancer Screening Trial (DLCST),^15^ there was an annual clinical exam and sputum cytology in both screening and control groups, but no imaging. In four other trials, Nederlands-Leuvens Longkanker Screenings Onderzoek (NELSON),^16^ Italian Lung Cancer Screening Trial (ITALUNG),^17^ Multicentre Italian Lung Detection Trial (MILD)^18^ and German Lung Cancer Screening Intervention (LUSI),^19^ smoking cessation was offered to participants in both groups, but no diagnostic procedures were offered to participants in the control group, as was true in the UKLS study.^2^

Recruitment began in 2000 for the earliest trial (LSS) and in 2011 for the most recent (UKLS). Participants were randomized to the CT screening group or the control group in roughly equal numbers in all trials except MILD, where the larger screening group was divided into those offered annual or biannual screening. In NELSON, four rounds of screening were separated by three progressively longer intervals (1 year, 2 years, 2.5 years); all other multi-round trials had annual screening. The number of screening rounds ranged from one (UKLS) to five (three trials), except that in one trial (MILD), the group with annual screening had up to seven rounds. High participation rates were the norm, with all but one trial (ITALUNG) having participation rates of at least 95%, although it should be noted that this participation rate was the percentage of people who had already agreed to participate in a screening trial.

LC mortality results were obtained from each trial's most recent report and are summarized in Table 3. The NLST, by far the largest trial by number of participants, had the longest FU data, with a median of 12.3 years.^1^ As discussed above, there was almost no increase in the number of DA since their initial report,^10^ since further FU added 791 deaths observed in the screening group and 793 in the control group, bringing the RR closer to 1.00 (0.928) than in previous reports. Altogether, 1804 deaths from LC were observed in the nine trials’ control groups, with 1624 LC deaths in the screening groups. PY of FU were similar in screening and control groups in all trials except MILD. LC mortality rates in the control groups varied from 19 per 10,000 PY to 54 per 10,000 PY, probably because admission criteria (based on age, sex, smoking history and, for ex-smokers, time since cessation) were different from trial to trial. LC mortality rates were lower in screened groups than in control groups, with non-statistically significant exceptions in two small trials. The overall RR, weighted by PYs of FU, was 0.89, for a mortality reduction of 11%.

**Table 3.** Most recent lung cancer mortality results from low-dose CT trials.

	Control			Screening			LC mort. Ratio (RR or HR)	Most recent report	Average FU for LC mort., median or mean (years)
Study	LC deaths	PY FU	LC mort. rate /1000 PY^a^	LC deaths	PY FU	LC mort. rate /1000 PY			
LSS	26	8384	3.10	32	8339	3.84	1.23	Doroudi et al., 2018	5.2
DANTE	55	10,104	5.44	59	10,875	5.43	0.99	Infante et al., 2015	8.35
NLST	1236	329,447.3	3.75	1147	329,348.7	3.48	0.92	NLST Res. Team, 2019	12.3
All CT vs CXR	1317	347,935.3	3.789	1238	348,562.7	3.55	0.93		
NELSON	263	81,633	3.22	205	81,967	2.50	0.77	De Koning et al., 2020	10
DLCST	38	19,547	1.94	39	19,439	2.01	1.03	Wille et al., 2015	9.47
ITALUNG	60	14,247	4.21	43	14,658	2.93	0.69	Paci et al., 2017	8.5
MILD	40	16,210	2.47	40	23,083	1.73	0.70	Pastorino et al., 2019	10
LUSI	40	17,802	2.25	29	17,855	1.62	0.72	Becker et al., 2019	8.8
UKLS	46	13,921.6	3.30	30	14,071.4	2.13	0.64	Field et al., 2021	7.3
All CT vs no CXR	487	163,361.0	2.96	386	171,073.6	2.26	0.76		
Total	1804	511,296.3	3.518	1624	519,636.3	3.13	0.88		

CXR: chest X-ray; RR: rate ratio, dividing lung cancer (LC) mortality rate in screening group by rate in control group; HR: hazard ratio; mort.: mortality; FU: follow-up; LSS: Lung Screening Study. ^a^PY: person-years; average LC mortality rates for three groupings are weighted by PYof FU for mortality in screening group, since this result is used for calculating expected deaths in the screening group.

Based on our reasoning that much of the FU data is noise and not signal, we posit that rate difference may be a preferable statistic that avoids this bias. We calculate here the number of LC deaths expected in the screening groups, adjusting for the difference in person-time in each trial's screening and control groups. Table 4 summarizes expected and observed LC deaths in the screening groups, along with DA and numbers of invitations to screening.

**Table 4.** Calculation of deaths averted and invitations or screens.

Study	Screening PY FU	Control LC mortality rate^a^	Screening expected LC deaths^b^	Screening, observed LC deaths	Deaths averted (95% CI)	Invitations to screening	Invitations /LC death averted
LSS	8339.0	3.10	25.9	32	−6.1	3027	−493
DANTE	10,875.0	5.44	59.2	59	0.2	6178	31,384
NLST	329,348.7	3.75	1235.6	1147	88.6	79,080	892
**All** CT vs CXR	348,562.7	3.79	1320.7	1238	**82.7 (−16.4, 181.8)**	88,285	**1068**
NELSON	81,967.0	3.22	264.1	205	59.1	28,455	482
DLCST	19,439.0	1.94	37.8	39	−1.2	10,260	−8480
ITALUNG	14,658.0	4.21	61.7	43	18.7	6386	341
MILD	23,083.0	2.47	57.0	40	17.0	13,008	767
LUSI	17,855.2	2.25	40.1	29	11.1	9886	889
UKLS	14,071.4	3.30	46.5	30	16.5	1981	120
**All** CT vs no CXR	171,073.6	2.96	507.2	386.0	**121.2 (63.3, 179.1)**	69,976	**577**
**TOTAL**	519,636.3	3.52	1827.9	1624	**203.9 (89.1, 318.6)**	158,261	**776**

CXR: chest X-ray; PY: person-years; FU: follow-up; CI: confidence interval; LSS: Lung Screening Study. ^a^Average rates for three groupings are weighted by PY of FU for mortality in screening group, since this result is used for calculating expected deaths in the screening group. ^b^applying control arm LC rates.

After the small adjustment for the slightly larger number of persons in trials’ screening groups, we obtain 1828 expected deaths in the screening groups, rather than the 1624 observed, resulting in a difference of 204 DA by screening. These gains were obtained by 157,199 invitations to CT screening. Dividing the number of invitations by the number of DA, 776 invitations were required to avert one death (“intention to screen” analysis). Because of the high rates of participation rates observed in the trials, per protocol analysis looking at the number of actual screens performed provided only small increases in screening's performance, of the order of 5%. In the three older trials comparing CT to CXR, 83 deaths were averted by 88,285 invitations, with 1068 invitations required to avert one death. In the six more recent trials with no screening in the control group, 121 deaths were averted by 69,976 invitations, i.e. 577 invitations to avert one death.

Screening can be expected to avert more deaths when the screening period is longer (more rounds), and when the underlying incidence rates are higher (more cases arising per round). Trial-specific underlying incidence rates can be estimated using the incidence rate observed in each control group, provided that no screening was offered to the control group. This is important if one wishes to extrapolate from the performance of screening, as observed in trials, to project anticipated performance in a program that may have different criteria for screening and thus a different undisturbed LC incidence rate. It is also important for those individuals whose risk differs from the screening trial average. In the six trials that had control groups without screening, average LC incidence rates varied between 2.7 and 5.5 cases per 1000 PYs, with an overall average of 4.4 cases per 1000 PYs.

## Discussion

We have shown that a total of 204 deaths were averted in nine trials, or 776 invitations to avert one death. The six more recent trials are better suited to estimating LC screening's benefits: they present the full contrast between CT screening and no screening, they represent more recent CT screening techniques, and they allow for estimating the underlying LC risk of participants. In these trials, 121 deaths were averted (95% CI 63-179), with 577 invitations to avert one death (95% CI 368-1042). Although a person's risk will be different (generally increasing) at the time of each round of screening, it is fair to say that a program with an average risk level that applied in these six trials will avert one LC death for every 23 persons who are invited 25 times to an annual screen (i.e. 577/25).

Analogous reasoning applies to an individual, whose risk will not usually be the same as the average risk of the population invited to the same program. For the calculation of an individual's anticipated benefit from screening, a linear adjustment to their risk level should be made, based on their estimated risk of LC. Numerous online calculators are available for establishing risk of LC, sometimes as the criterion for participation in LC screening programs, rather than using fixed cut-offs like age and pack-years of smoking.^20,21^

From the best available trial data (the six trials without screening in the control group), we found that 121 deaths were averted with 507 LC deaths expected, and 386 were observed in patients invited to CT screening, representing a 24% reduction. However, this 24% reduction has very different implications for patients with differing levels of risk. The annual rate of 4.4 LC cases/1000 persons observed in the control groups of these trials represents a risk of about 2.6% of developing LC in the next 6 years, and this average trial population risk can be compared, using a risk assessment tool like the calculator^b^ proposed by Tammemägi,^20^ to the LC risk of different patients who might be invited to a screening program. Assuming an age of onset of smoking of 15, a high school level of education, no personal history of lung disease or cancer, and no family history of LC, we find the following 6-year risks of being diagnosed with LC:

1. a 56-year-old ex-smoker of 20 cigarettes/day for 31 years who quit 10 years ago: 0.7%
2. a 58-year-old ex-smoker of 20 cigarettes/day for 33 years who quit 5 years ago: 1.3%
3. a 59-year-old smoker of 20 cigarettes/day for 44 years: 2.6%
4. a 62-year-old smoker of 40 cigarettes/day for 47 years: 5.5%
5. a 69-year-old smoker of 40 cigarettes a day for 54 years: 11.2%.

These five patients span a huge range of risk levels, going from the 56-year-old ex-smoker with four times less risk than the trial average, up to the 69-year-old smoker with risk that it four times higher than the average; even lower and higher risk levels are possible. Although trials may have shown that, on average, 577 invitations were required to avert one death, this will be a poor estimate for most participants, since patients like the one with the lowest risk among the five examples would need four times more invitations to screening to avert one death, whereas patients like the highest risk patient would require four times less.

This approach assumes that the benefits of LC screening scale linearly with LC cancer risk. While we feel that this assumption is logical, it does involve an additional step in calculating a patient's anticipated benefit from screening. We note that the assumption of linearity is already implicitly made when RRs are pooled, in populations with different underlying risk, and when RR-based benefits are presented to individuals who have different levels of LC risk.

As with analyses pooling trials and their RR measures, this analysis ignores what is sure to be a different effect in first and subsequent rounds of screening, since the first round also picks up some cases that will have been detectable for longer than the interval between screens. We see no obvious way to quantify this differential effect, outside of a new trial methodology that could randomize some participants to a single round of screening (such as UKLS) while randomizing another group to two rounds.

With FU beyond the time point where the number of DA has plateaued, the RR will increasingly approach one, while the number of DA will not change. However, for the calculation of confidence intervals, FU that continues for too long will widen the confidence intervals, since they are a function of the square root of the total number of deaths. It thus remains important to determine when the number of DA plateaus, and it would be useful for trial reports to provide the numbers of DA at different time points, annually for instance.

Calculations of the number of screens required to avert one death will not be useful when the number of DA is negative (leading to a negative number of screens to avert a death), nor for individual trials where the number of DA is very small, or when confidence intervals include zero DA. Such calculations are probably better suited to large trials or collections of trials with significant positive results.

Finally, we have given estimates using the number of people invited to screening, based on the “intention to screen” principle. This will underestimate the performance that would be possible in people actually screened. Because participation was high (95%) in the LC screening trials, the underestimate is small, and so we have presented only an intention to screen analysis, but for cancer screening trials with lower participation rates, one should consider calculating per protocol effectiveness for the purposes of informed consent.

Whether it is for LC screening or screening of other conditions, we believe that the calculation of the absolute number of DA, along with the ratios of screening invitations and screening episodes to DA, avoids the bias to the null associated with use of ratio statistics like RR and HR. We have shown that the calculation of this metric is feasible using existing trial data and can be easily adjusted for individuals or populations with risk levels different to those that prevailed in trials. In comparison with RRs, the absolute number of DA may provide a more intuitive summary of the expected efficacy of a screening program while allowing a personalized estimate of the expected benefits of a screening program to an individual who is contemplating screening.

## Notes

a. All CT screening trials and programs use low-dose techniques with mean effective doses of 1.5 mSv or less, compared to an average of about 7 mSv for conventional CT. See NCCN Clinical Practice Guidelines in Oncology (NCCN Guidelines) Lung Cancer Screening Version 2.2024—18 October 2023.

b. Lung Cancer Risk Prediction Calculator excluding the race/ethnicity predictor (PLCOm2012noRace).

## References

[1] National Lung Screening Trial Research Team. Lung cancer incidence and mortality with extended follow-up in the national lung screening trial. J Thorac Oncol 2019; 14: 1732–1742. Epub 2019 Jun 28. PMID: 31260833; PMCID: PMC6764895. 31260833 10.1016/j.jtho.2019.05.044 PMC6764895

[2] Field JK Vulkan D Davies MPA , et al. Lung cancer mortality reduction by LDCT screening: UKLS randomised trial results and international meta-analysis. Lancet Reg Health Eur 2021; 10: 100179. 34806061 10.1016/j.lanepe.2021.100179 PMC8589726

[3] Hanley JA Deck W Kochoedo M . Underestimating the lung cancer mortality reductions produced by low-dose CT screening. Lancet Reg Health Eur 2021; 11: 100256. PMID: 34778862; PMCID: PMC8577131. 34778862 10.1016/j.lanepe.2021.100256 PMC8577131

[4] Jonas DE Reuland DS Reddy SM , et al. Screening for lung cancer with low-dose computed tomography: updated evidence report and systematic review for the US preventive services task force. JAMA 2021; 325: 971–987. PMID: 33687468. 33687468 10.1001/jama.2021.0377

[5] Passiglia F Cinquini M Bertolaccini L , et al. Benefits and harms of lung cancer screening by chest computed tomography: a systematic review and meta-analysis. J Clin Oncol 2021; 39: 2574–2585. Epub 2021 Jun 2. Erratum in: J Clin Oncol. 2021 Oct 1;39(28):3192-3193. PMID: 34236916. 34236916 10.1200/JCO.20.02574

[6] Sadate A Occean BV Beregi JP , et al. Systematic review and meta-analysis on the impact of lung cancer screening by low-dose computed tomography. Eur J Cancer 2020; 134: 107–114. Epub 2020 Jun 2. PMID: 32502939. 32502939 10.1016/j.ejca.2020.04.035

[7] Huang KL Wang SY Lu WC , et al. Effects of low-dose computed tomography on lung cancer screening: a systematic review, meta-analysis, and trial sequential analysis. BMC Pulm Med 2019; 19: 126. PMID: 31296196; PMCID: PMC6625016. 31296196 10.1186/s12890-019-0883-x PMC6625016

[8] Hoffman RM Atallah RP Struble RD , et al. Lung cancer screening with low-dose CT: a meta-analysis. J Gen Intern Med 2020; 35: 3015–3025. Epub 2020 Jun 24. PMID: 32583338; PMCID: PMC7573097 32583338 10.1007/s11606-020-05951-7 PMC7573097

[9] Baker SG Kramer BS Prorok PC . Early reporting for cancer screening trials*.* J Med Screen 2008; 15: 122–129. 18927094 10.1258/jms.2008.007058 PMC2586667

[10] National Lung Screening Trial Research Team, Aberle DR Adams AM Berg CD , et al. Reduced lung-cancer mortality with low-dose computed tomographic screening. N Engl J Med 2011; 365: 395–409. 21714641 10.1056/NEJMoa1102873 PMC4356534

[11] Pinsky PF Church TR Izmirlian G , et al. The national lung screening trial: results stratified by demographics, smoking history, and lung cancer histology. Cancer 2013; 119: 3976–3983. Epub 2013 Aug 26. 24037918 10.1002/cncr.28326 PMC3936005

[12] Duffy SW Smith RA . A note on the design of cancer screening trials. J Med Screen 2015; 22: 65–68. 25767104 10.1177/0969141315577847

[13] Gohagan JK Marcus PM Fagerstrom RM , et al. Final results of the lung screening study, a randomized feasibility study of spiral CT versus chest X-ray screening for lung cancer. Lung Cancer 2005; 47: 9–15. PMID: 15603850. 15603850 10.1016/j.lungcan.2004.06.007

[14] Infante M Cavuto S Lutman FR , et al. Long-term follow-up results of the DANTE trial, a randomized study of lung cancer screening with spiral computed tomography. Am J Respir Crit Care Med 2015; 191: 1166–1175. PMID: 25760561. 25760561 10.1164/rccm.201408-1475OC

[15] Wille MM Dirksen A Ashraf H , et al. Results of the randomized danish lung cancer screening trial with focus on high-risk profiling. Am J Respir Crit Care Med 2016; 193: 542–551. PMID: 26485620. 26485620 10.1164/rccm.201505-1040OC

[16] de Koning HJ van der Aalst CM de Jong PA , et al. Reduced lung-cancer mortality with volume CT screening in a randomized trial. N Engl J Med 2020; 382: 503–513. Epub 2020 Jan 29. PMID: 31995683. 31995683 10.1056/NEJMoa1911793

[17] Paci E Puliti D Lopes Pegna A , et al. Mortality, survival and incidence rates in the ITALUNG randomised lung cancer screening trial. Thorax 2017; 72: 825–831. Epub 2017 Apr 4. PMID: 28377492. 28377492 10.1136/thoraxjnl-2016-209825

[18] Pastorino U Sverzellati N Sestini S , , et al. Ten-year results of the multicentric Italian lung detection trial demonstrate the safety and efficacy of biennial lung cancer screening. Euro J Cancer 2019; 118: 142–148. 10.1016/j.ejca.2019.06.009 PMC6755135 31336289

[19] Becker N Motsch E Trotter A , et al. Lung cancer mortality reduction by LDCT screening-results from the randomized German LUSI trial. Int J Cancer 2020; 146: 1503–1513. 31162856 10.1002/ijc.32486

[20] Tammemägi MC . Selecting lung cancer screenees using risk prediction models-where do we go from here. Transl Lung Cancer Res 2018; 7: 243–253. PMID: 30050763; PMCID: PMC6037970. 30050763 10.21037/tlcr.2018.06.03 PMC6037970

[21] Pasquinelli MM Tammemägi MC Kovitz KL , et al. Risk prediction model versus United States preventive services task force lung cancer screening eligibility criteria: reducing race disparities. J Thorac Oncol 2020; 15: 1738–1747. Epub 2020 Aug 18. PMID: 32822843. 32822843 10.1016/j.jtho.2020.08.006

